# Evaluating the effectiveness of distortion self‐correction for CEST‐EPI

**DOI:** 10.1002/mrm.70048

**Published:** 2025-08-29

**Authors:** Yang Liu, Se Weon Park, Lok Hin Law, Kexin Wang, Licheng Ju, Jiadi Xu, Kannie W. Y. Chan, Jianpan Huang

**Affiliations:** ^1^ Department of Biomedical Engineering City University of Hong Kong Hong Kong China; ^2^ Hong Kong Centre for Cerebro‐Cardiovascular Health Engineering (COCHE) Hong Kong China; ^3^ Department of Biomedical Engineering Johns Hopkins University Baltimore Maryland USA; ^4^ F.M. Kirby Research Center for Functional Brain Imaging Kennedy Krieger Research Institute Baltimore Maryland USA; ^5^ Russell H. Morgan Department of Radiology and Radiological Science The Johns Hopkins University School of Medicine Baltimore Maryland USA; ^6^ State Key Laboratory of Terahertz and Millimeter Waves City University of Hong Kong Hong Kong China; ^7^ Tung Biomedical Sciences Centre (TBSC) City University of Hong Kong Hong Kong China; ^8^ City University of Hong Kong Shenzhen Research Institute Shenzhen China; ^9^ Department of Diagnostic Radiology, Li Ka Shing Faculty of Medicine The University of Hong Kong Hong Kong China

**Keywords:** ΔB_0_ map, CEST, distortion, EPI, self‐correction

## Abstract

**Purpose:**

EPI is a fast acquisition sequence, but suffers from geometric distortion because of B_0_ field inhomogeneity. This study aims to evaluate the effectiveness of using ΔB_0_ map generated from single‐shot CEST‐EPI to achieve distortion self‐correction (DISC).

**Methods:**

CEST MRI usually requires B_0_ correction during postprocessing, and the ΔB_0_ map can be calculated directly from Z‐spectra without extra scan. We propose to use the ΔB_0_ map to correct the geometry distortion induced by B_0_ field inhomogeneity in CEST‐EPI. The effectiveness of DISC strategy for CEST‐EPI was evaluated on a creatine phantom, healthy and tumor mice at 3 T, and AQP4 heterozygotes mice at 11.7 T. For both the original CEST images and the generated CEST contrast maps, the spatial improvement was first confirmed by the visual comparison and then quantitatively assessed by the structural similarity index measure (SSIM) comparison together with correlation analysis.

**Results:**

DISC‐CEST‐EPI showed higher SSIM and spatial consistency compared to CEST‐EPI when using CEST‐rapid acquisition with relaxation enhancement as a reference in vitro and in vivo at 3 T. For in vivo experiments at 11.7 T, SSIM values of amide proton transfer and relayed nuclear Overhauser effects maps of DISC‐CEST‐EPI were slightly higher than CEST‐EPI and comparable with field‐mapping and top‐up.

**Conclusion:**

DISC‐CEST‐EPI can correct the distortion in CEST‐EPI at 3 T, leading to improved SSIM and CEST quantification. This approach has the potential to enhance image quality and improve diagnostic accuracy for single‐shot CEST‐EPI at especially low‐field MRI. However, the performance of DISC‐CEST‐EPI is limited at 11.7 T, and further advancements are necessary to enhance its functionality at ultra‐high fields.

## 1 INTRODUCTION

CEST MRI is a promising molecular imaging technique that can detect low‐concentration molecules via the exchange between the water protons and exchangeable solute protons.^1^, ^2^ It has been widely used to image many endogenous contrasts, such as proteins or peptides (amide proton transfer [APT]),^3^, ^4^ glutamate (gluCEST),^5^, ^6^ creatine (CrCEST),^7^, ^8^, ^9^ and glucose (glucoCEST).^10^, ^11^, ^12^, ^13^, ^14^, ^15^ In addition to the direct CEST effects downfield in water saturation spectrum (Z‐spectrum), relayed nuclear Overhauser effects (rNOE) upfield from the water signal have also drawn much attention over the past years.^16^, ^17^, ^18^, ^19^, ^20^, ^21^ Therefore, by using the exchangeable protons in an increasing library of biomolecules and compounds, CEST MRI has great potential in disease diagnosis and treatment monitoring. However, some limitations of CEST MRI have hindered its widespread clinical application. For example, CEST suffers from the problem of long scan time as a series of images at different saturation frequency offsets are acquired for one CEST dataset. Therefore, many studies have explored fast‐acquisition sequences for accelerating CEST MRI.^22^, ^23^, ^24^, ^25^, ^26^, ^27^ Typically, a CEST sequence consists of a saturation module and an acquisition module. The acquisition module theoretically can be most conventional readout sequences. Currently, many readout sequences have been incorporated into CEST sequences, such as EPI,^3^, ^28^, ^29^, ^30^, ^31^ gradient echo (GRE),^22^, ^32^, ^33^, ^34^, ^35^, ^36^, ^37^ fast/turbo spin echo (FSE/TSE) or rapid acquisition with relaxation enhancement (RARE),^20^, ^21^, ^38^, ^39^, ^40^, ^41^, ^42^, ^43^, ^44^, ^45^ gradient and spin echo (GRASE),^46^, ^47^ optimized TSE with variable flip angle evolutions (SPACE),^45^ periodically rotated overlapping parallel lines enhanced reconstruction (PROPELLER),^24^, ^48^ and stack‐of‐spirals (SOS) gradient echo readout.^49^ FSE/TSE/RARE is a commonly used imaging approach that is insensitive to susceptibility distortion and has a high SNR, but the scan time is relatively long especially when whole‐region coverage is requested. Single‐shot EPI is a fast acquisition alternative for CEST, but suffers from image distortion caused by B_0_ field inhomogeneity and low SNR because of T_2_ attenuation. The image distortion of EPI is mainly caused by the phase error accumulated during the long readout window.^50^ This distortion typically appears as pixel shifts along the phase encoding (PE) direction, which are proportional to the phase error accumulation determined by the B_0_ field inhomogeneity and effective echo spacing (ESP).

Two commonly used EPI distortion correction methods are field‐mapping method^50^, ^51^ and the top‐up method (also known as blip up/down method or gradient reversal method).^28^, ^52^ The field‐mapping method requires additional acquisition of B_0_ field inhomogeneity using ΔB_0_ mapping method such as GRE with varied TE. The B_0_ field inhomogeneity is then used to generate the displacement map (pixel shift map) for correcting distorted EPI images by coordinate reposition and linear interpolation in the image domain. The top‐up method requires the acquisition of two sets of EPI images with opposite polarities along the PE direction to estimate the displacement map. Therefore, both methods require additional acquisitions.

In CEST imaging, the B_0_ field inhomogeneity map can be generated from Z‐spectrum by data interpolation and minimum search, and typically is used to correct Z‐spectrum.^51^, ^53^, ^54^, ^55^ Here, we evaluated the effectiveness of using the field map generated by the Z‐spectrum to achieve the distortion self‐correction (DISC) for single‐shot CEST‐EPI without additional acquisition of a ΔB_0_ field map. The effectiveness of DISC strategy for CEST‐EPI was evaluated on a creatine phantom and in vivo healthy and tumor mouse brains at 3 T, using CEST‐RARE as a reference. For both the original CEST images and the generated CEST contrast maps, the spatial improvement was first confirmed by the visual comparison and then quantitatively assessed by the structural similarity index measure (SSIM)^56^ comparison together with correlation analysis. Furthermore, we applied DISC‐CEST‐EPI at 11.7 T to test its reliability under a high field strength, comparing its performance with traditional field‐mapping and top‐up methods.

## 2 METHODS

### 2.1 Phantom experiments at 3 T

Creatine phantoms with different concentrations (i.e., 5, 10, 20, 30, 40, and 50 mM) were prepared by dissolving the creatine (Sigma‐Aldrich) in deionized (DI) water titrated to pH 7.3. The prepared six creatine samples together with one DI water sample were separately put into seven 0.6‐mL microcentrifuge tubes for MRI experiments.

CEST MRI experiments were performed on a horizontal bore 3 T Bruker BioSpec system (Bruker). The creatine phantom was scanned under 37°C maintained by a warm air pipe connecting to a water heating system (Thermo Fisher Scientific). Random B_0_ inhomogeneity was introduced before conducting the CEST imaging. CEST data from axial view of creatine phantom was acquired. CEST MRI sequence was a continuous‐wave (CW) saturation module followed by EPI or RARE as a readout module. The CEST MRI parameters were set as follows: saturation power (B_1_) = 0.8 μT, saturation time (*t*_sat_) = 3 s, TR = 6 s, TE = 43 ms, FOV = 35 × 35 mm^2^, matrix size = 96 × 96, bandwidth = 250 kHz (CEST‐EPI), RARE factor = 24 (CEST‐RARE). Hence, the scan time for one image was 6 s and 24 s for CEST‐EPI and CEST‐RARE, respectively. The saturation frequency offset was swept from −10 to 10 ppm, with a 0.1 ppm step size between −4 and 4 ppm and a 1 ppm step size between −4/4 and −10/10 ppm. Additional CEST images at frequency offsets of ±1.75 ppm, ±1.85 ppm, ±1.95 ppm, ±2.05 ppm, and ±2.15 ppm were acquired. Three M_0_ images with saturation frequency offset at 300 ppm were acquired and last two of them were averaged for Z‐spectrum normalization. Therefore, the total image number for each CEST dataset was 106, resulting in a scan time of 10.6 min for CEST‐EPI and 42.4 min for CEST‐RARE.

### 2.2 Mouse experiments at 3 T

Mouse experiments at 3 T were approved by the Animal Research Ethics Sub‐Committee of Research Committee and followed the institutional guidelines of Institutional Laboratory Animal Research Unit of City University of Hong Kong. Six C57BL/6 mice (male, 2 months old) and six NOD‐SCID mice (female, 2 months old) with U87 tumor implantation were used in this study. For tumor implantation, mouse (NOD‐SCID) was anesthetized by 2% isoflurane in concentrated oxygen before being positioned on a stereotactic frame. The isoflurane concentration was then changed to 1.5% to maintain anesthesia. Bregma was revealed by creating a 1 cm incision on mice scalp along the sagittal orientation and was set as the coordinate origin. A small burr hole was drilled on the skull at 0.2 mm anterior and 2 mm lateral from the right of the bregma using a syringe needle. U‐87 MG glioma cells with density of 0.5 M/3 μL were injected into mouse brain at 0.2 mm anterior, 2.0 mm right‐lateral, and 3.8 mm below the bregma using a Hamilton syringe at a rate of 0.3 μL/min. The needle of the Hamilton syringe was kept at the location for 5 min after injection and was slowly withdrawn. The burr hole was sealed with bone wax before suturing the scalp. The body temperature of mouse was maintained at 37°C. CEST MRI was performed 2 to 3 weeks after tumor implantation.^57^

CEST MRI experiments were performed on a horizontal bore 3 T Bruker BioSpec system (Bruker). A 40‐mm volume transceiver coil was used for the scan of creatine phantom and mice 1 to 8 (Table 1). An 82‐mm quadrature volume resonator as a transmitter and a single surface coil as a receiver were used for the scan of mice 9 to 12 (Table 1). For CEST MRI of mice, anesthesia was induced and maintained with 2% and 1% to 1.5% isoflurane in oxygen, respectively, using an Animal Anesthesia Machine (RWD Life Science). Mice were placed on animal bed with head positioned using a bite bar. During the MRI scan, the mouse body temperature was maintained with a water‐heated pad connecting to the water heating system and the mouse respiratory was continuously monitored by an Animal Monitoring and Gating System (SA Instruments). CEST data from axial view of mouse head was acquired. The CEST MRI sequence and parameters were the same as those used in the phantom experiment, with the following exceptions: TR = 5 s, FOV = 20 × 20 mm^2^. The saturation frequency offsets were swept from −20 to 20 ppm, with an increment of 0.25 ppm between −8 and 8 ppm, an increment of 1 ppm between −8/8 and −10/10 ppm, and an increment of 5 ppm between −10/10 to −20/20 ppm. Three M_0_ images with saturation frequency offset at 300 ppm were acquired and last two of them were averaged for Z‐spectrum normalization. Therefore, the total image number for each CEST dataset was 76, resulting in a scan time of 6.33 min for CEST‐EPI and 25.33 min for CEST‐RARE.

### 2.3 Mouse experiments at 11.7 T

Mouse experiment procedures at 11.7 T were approved by the Johns Hopkins University Animal Care and Use Committee. Three AQP4 heterozygotes mice (2 male, 1 female, 6–12 months old) were used. MRI experiments were performed on a horizontal bore 11.7 T Bruker Biospec system (Bruker). A 72‐mm quadrature volume resonator was used as a transmitter, and a 2 × 2 phased array was used as a receiver for animal MRI. Animals were anesthetized using 2% isoflurane in medical air, followed by 1% to 1.5% isoflurane for maintenance. The CEST MRI sequence and parameters were exactly the same as those used in the 3 T mouse experiments, with exceptions that TR = 6 s, TE = 15.9 ms (CEST‐EPI), TE = 44 ms (CEST‐RARE), and RARE factor = 48 (CEST‐RARE). Two CEST‐EPI sequences with opposite phase encoding, one forward and one reverse along y direction, were scanned for top‐up method. Two GRE acquisitions using FLASH sequence with TE = 3 ms and 6 ms were scanned for additional ΔB_0_ map calculation for field‐mapping method. The total scan time is 7.6 min for CEST‐EPI, 15.2 min for CEST‐RARE, and 0.64 min for FLASH.

### 2.4 Distortion self‐corrected CEST‐EPI

**FIGURE 1:**
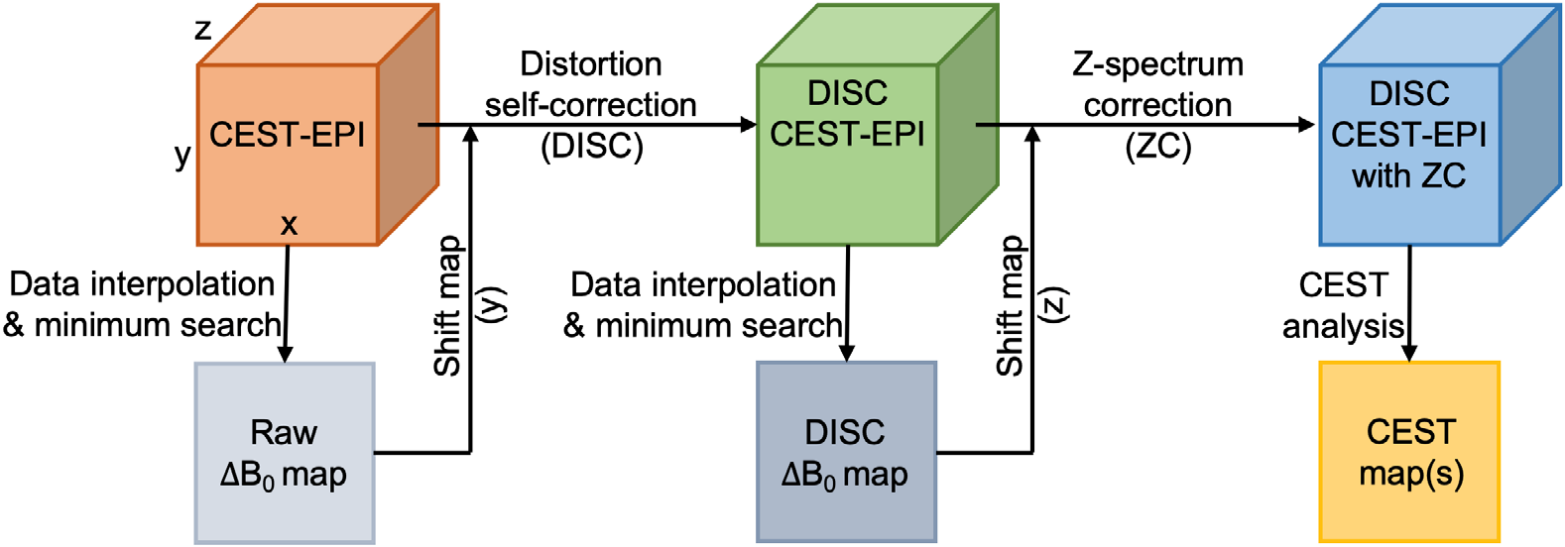
Schematic diagram of the distortion self‐correction (DISC) flow for single‐shot CEST‐EPI. First, the ΔB_0_ map was generated from CEST‐EPI and converted into pixel shift map. Second, the pixel shift map was used to correct geometric distortion by interpolating and shifting the signal intensity of each phase encoding (PE) line to generate DISC‐CEST‐EPI dataset. Finally, the DISC ΔB_0_ map was generated from the DISC‐CEST‐EPI dataset and used for Z‐spectrum correction (ZC). Frequency encoding is along *x* direction, PE is along y direction.

CEST‐EPI processing procedures including the proposed distortion self‐correction is illustrated in Figure 1. The distortion correction was only considered along the PE direction (*y*), as the shift in frequency encoding (FE) direction (*x*) is negligible.^50^ First, the ΔB_0_ map was generated by data interpolation and minimum search of the Z‐spectrum for each CEST‐EPI dataset. The ΔB_0_ map was then converted into a pixel shift map along the PE direction using the equation:

(1) $$∆r_{\mathrm{pe}}=\gamma \cdot ∆B_{0}\cdot N_{\mathrm{pe}}\cdot \mathrm{ESP},$$

where γ is gyromagnetic ratio, Δ*B*_0_ is field inhomogeneity, $N_{\mathrm{pe}}$ is PE number, and *ESP* is effective echo spacing. Second, the pixel shift map was used to correct distortion by interpolating and shifting the signal intensity of each PE line to obtain the DISC‐CEST‐EPI dataset. The relationship between pixel positions with (*y*) and without (*y*_dist_) distortion can be expressed as:

(2) $$y_{\text{dist}}=y+∆r_{\mathrm{pe}}.$$

Finally, the DISC ΔB_0_ map was generated using the DISC‐CEST‐EPI dataset and used for Z‐spectrum correction (ZC) by interpolating and shifting the signal intensity of each Z‐spectrum.^53^ After self‐correction, Z‐spectrum were obtained by the following equation^1^, ^58^:

(3) $$Z\left(∆\omega \right)=\frac{M_{\mathrm{sat}}\left(∆\omega \right)}{M_{0}},$$

where ∆ω represented the saturation frequency offset with respect to the water frequency at 0 ppm, $M_{\mathrm{sat}}\left(∆\omega \right)$ and $M_{0}$ were the steady‐state magnetization with saturation at ∆ω and without saturation (or with saturation at large ∆ω, such as 300 ppm here), respectively. CEST analysis was performed to obtain the CEST contrast. For creatine phantom, CEST map at 2 ppm was generated using the magnetization transfer ratio asymmetry (*MTR*_asym_) analysis^4^:

(4) $${\mathrm{MTR}}_{\text{asym}}\left(∆\omega \right)=Z\left(-2\;\mathrm{ppm}\right)-Z\left(2\;\mathrm{ppm}\right).$$

For mouse brain, the direct water saturation (DS) was first fitted using a Lorentzian function^59^, ^60^ to serve as a reference spectrum *Z*_ref_ (∆*ω*). This was followed by inverse Z‐spectrum analysis to generate the CEST contrasts at 3.5 ppm for the APT and at −3.5 ppm for the rNOE^61^:

(5) $${\text{CEST}}_{\mathrm{rex}}=\frac{1}{Z\left(∆\omega \right)}-\frac{1}{Z_{\mathrm{ref}}\left(∆\omega \right)}.$$

To quantitatively assess the correction effectiveness of the geometric distortion, SSIM values of both original images and CEST maps were calculated for CEST‐EPI and DISC‐CEST‐EPI with reference to CEST‐RARE.^56^

## 3 RESULTS

### 3.1 The 3 T in vitro creatine phantom results

**FIGURE 2:**
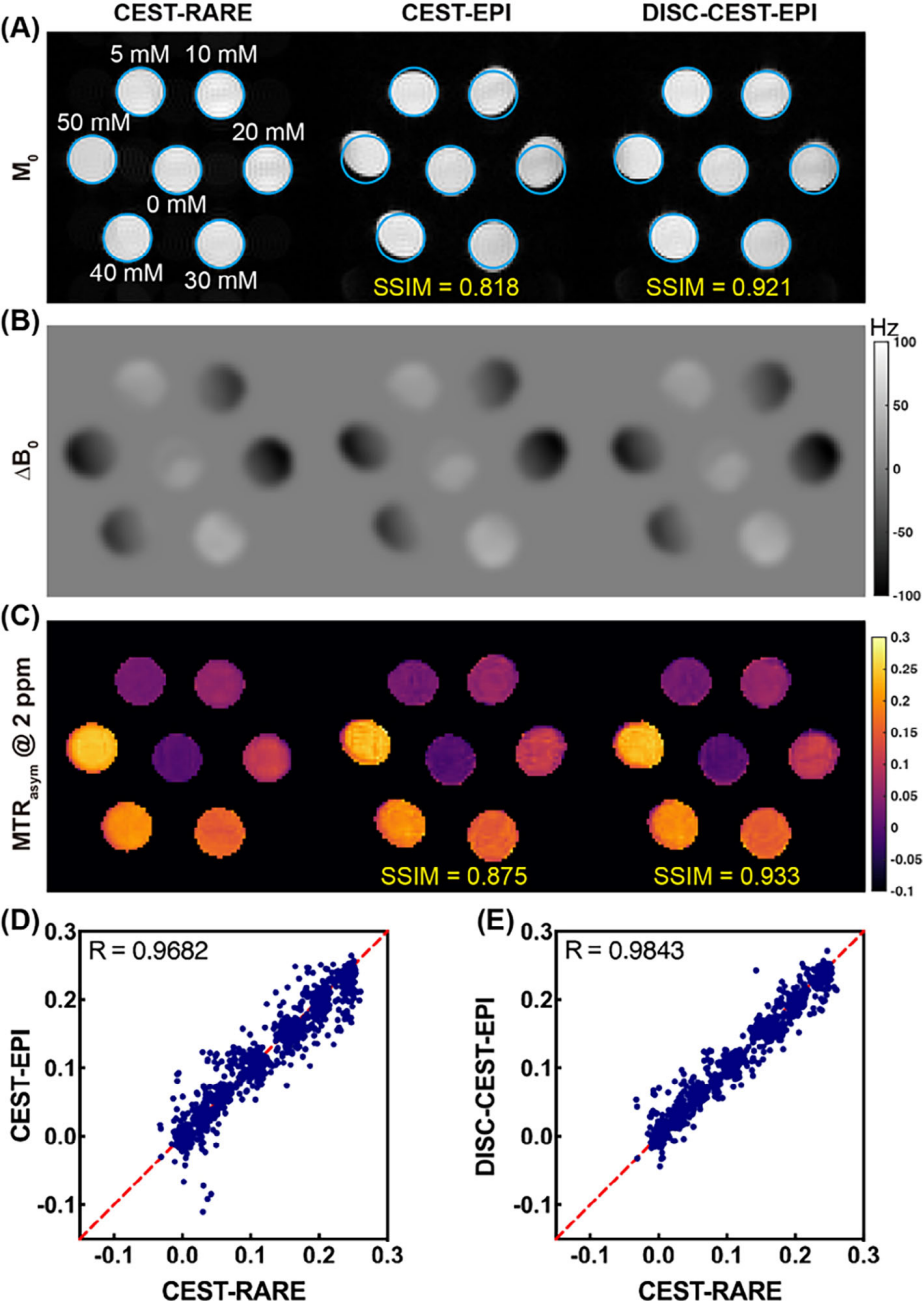
Comparison of CEST‐rapid acquisition with relaxation enhancement (RARE) with CEST‐EPI/distortion self‐correction (DISC)‐CEST‐EPI in a creatine phantom at 3 T. (A) Original CEST images (M_0_ images). (B) ΔB_0_ maps. (C) Creatine CEST maps. (D) Spatial correlation of creatine CEST values between CEST‐RARE and CEST‐EPI. (E) Spatial correlation of creatine CEST values between CEST‐RARE and DISC‐CEST‐EPI.

The CEST results of the creatine phantom acquired by CEST‐RARE and CEST‐EPI with and without correction are presented in Figure 2. All tubes with different concentrations of creatine were accurately captured by CEST‐RARE, as each tube appeared as a regular circle in the image (Figure 2A). However, the CEST‐EPI image acquired at the same slice exhibited severe geometric distortion for all tubes, resulting in large misalignments with the blue masks generated from the CEST‐RARE image. The SSIM of the entire CEST dataset was 0.818 when compared to the CEST‐RARE results. As expected, the distortion was transferred to the CrCEST map generated by the *MTR*_asym_ (Figure 2C), which showed an SSIM of 0.875. This distortion was primarily induced by the B_0_ field inhomogeneity, as it exhibited a similar pattern to the intensity of ΔB_0_ map (Figure 2B). Notably, this distortion was effectively corrected in DISC‐CEST‐EPI image, as all tubes aligned well with the blue masks, and the SSIM of the entire CEST dataset was enhanced to 0.921 (Figure 2A). The effectiveness of distortion correction was also evident in CrCEST map, which exhibited a significantly improved SSIM of 0.933 (Figure 2C). Furthermore, the correlation analysis results demonstrated that the CrCEST signals of DISC‐CEST‐EPI exhibited higher spatial consistency with CEST‐RARE compared to CEST‐EPI, with correlation coefficients of *R* = 0.9843 and *R* = 0.9682, respectively (Figure 2D,E).

### 3.2 The 3 T in vivo mouse brain results

**FIGURE 3:**
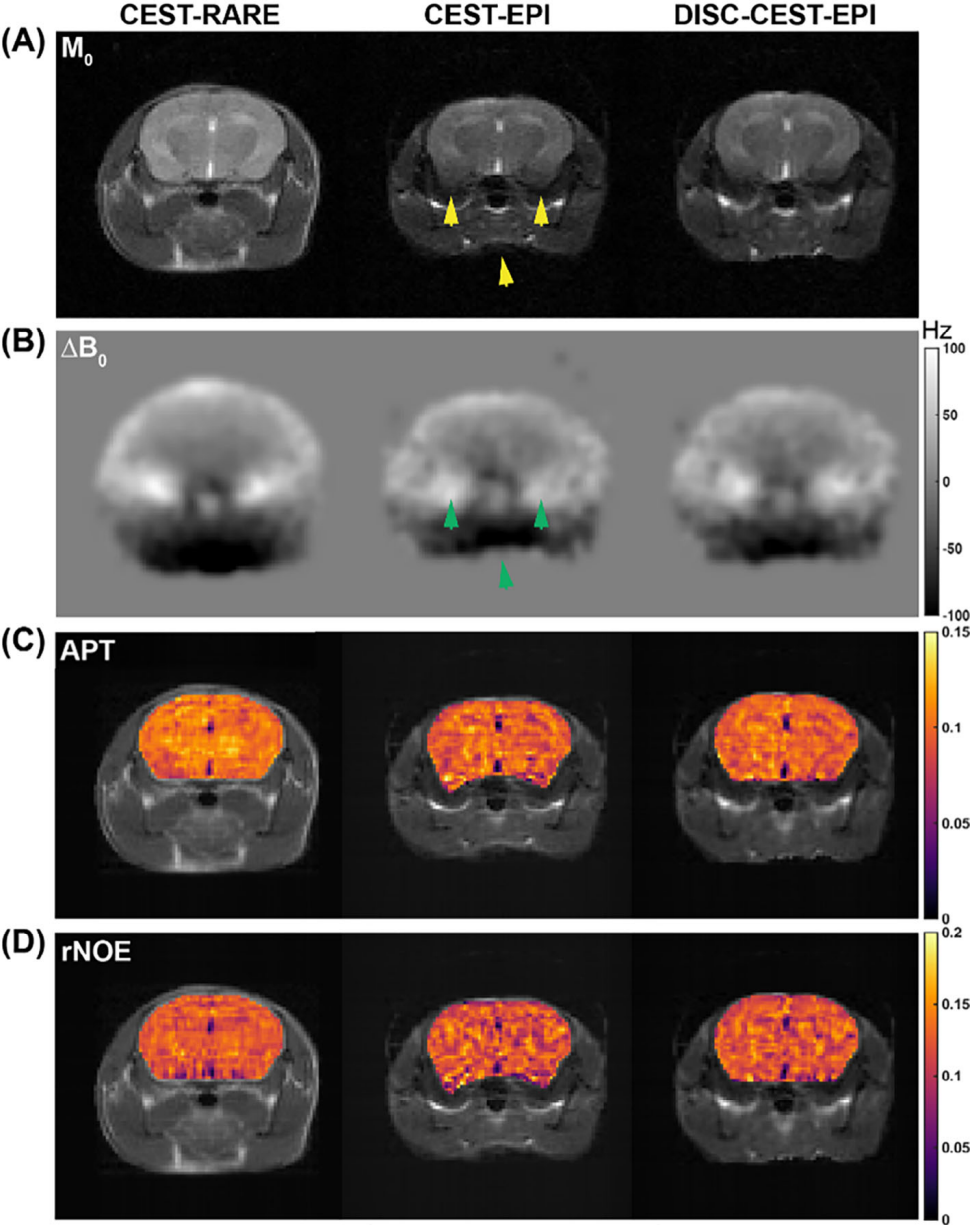
Comparison of CEST‐rapid acquisition with relaxation enhancement (RARE) with CEST‐EPI/distortion self‐correction (DISC)‐CEST‐EPI in a normal mouse brain at 3 T. (A) Original CEST images (M_0_ images). (B) ΔB_0_ maps generated from Z‐spectrum. (C) Amide proton transfer (APT) maps. (D) Relayed nuclear Overhauser effects (rNOE) maps. Phase encoding is along vertical view.

We first investigated the effectiveness of DISC‐CEST‐EPI in six normal C57BL/6 mice at 3 T. Figure 3 displays the CEST results of a normal mouse head acquired using CEST‐RARE and CEST‐EPI with and without correction. CEST‐RARE provided a clear image of the mouse head, including regions of brain and muscle (Figure 3A). However, the CEST‐EPI image from the same slice exhibited noticeable geometric distortion, particularly at interfaces such as the brain‐muscle and muscle‐air interfaces (indicated by yellow arrows in Figure 3A). Regions with significant ΔB_0_ inhomogeneity, as indicated by the green arrows in Figure 3B, were particularly affected by severe distortion. The distortion was also evident in the APT and rNOE maps (Figure 3C,D). Notably, the application of distortion self‐correction in DISC‐CEST‐EPI resulted in significant improvements in both the original CEST image (Figure 3A) and the CEST contrast maps (Figure 3C,D). The SSIM values for the six mice (Table 1) demonstrated improved image quality in all tested cases. Specifically, DISC‐CEST‐EPI improved the SSIM value of the entire CEST dataset from 0.634 ± 0.068 to 0.681 ± 0.069 (*p* = 0.0002). Moreover, the SSIM values of the APT and rNOE maps increased from 0.909 ± 0.014 and 0.894 ± 0.010 to 0.940 ± 0.012 and 0.929 ± 0.007, respectively (*p* < 0.0001).

**TABLE 1:** Comparison of SSIM values of CEST images and contrast maps for CEST‐EPI and DISC‐CEST‐EPI of normal mice and tumor mice at 3 T.

	Mouse no.	All CEST images		APT map		rNOE map	
		CEST‐EPI	DISC‐ CEST‐EPI	CEST‐EPI	DISC‐ CEST‐EPI	CEST‐EPI	DISC‐ CEST‐EPI
Normal	1	0.515	0.562	0.883	0.916	0.888	0.925
	2	0.624	0.682	0.909	0.943	0.878	0.919
	3	0.636	0.689	0.921	0.949	0.901	0.931
	4	0.630	0.666	0.920	0.945	0.891	0.925
	5	0.712	0.769	0.908	0.946	0.904	0.941
	6	0.688	0.717	0.913	0.938	0.902	0.930
	Mean	0.634	0.681	0.909	0.940	0.894	0.929
	SD	0.068	0.069	0.014	0.012	0.010	0.007
	*p* value	0.0002***		<0.0001****		<0.0001****	
Tumor	7	0.769	0.795	0.919	0.928	0.903	0.917
	8	0.726	0.751	0.944	0.955	0.938	0.953
	9	0.851	0.870	0.909	0.923	0.913	0.932
	10	0.816	0.843	0.903	0.940	0.903	0.941
	11	0.830	0.858	0.915	0.932	0.909	0.927
	12	0.806	0.844	0.888	0.919	0.877	0.908
	Mean	0.800	0.827	0.913	0.933	0.907	0.930
	SD	0.045	0.045	0.019	0.013	0.020	0.016
	*p* value	0.0001***		0.0082**		0.0024**	

**p ≤ 0.01; ***p ≤ 0.001; ****p ≤ 0.0001.

Abbreviations: APT, amide proton transfer; DISC, distortion self‐correction; rNOE, relayed nuclear Overhauser effects; SSIM, structural similarity index measure.

**FIGURE 4:**
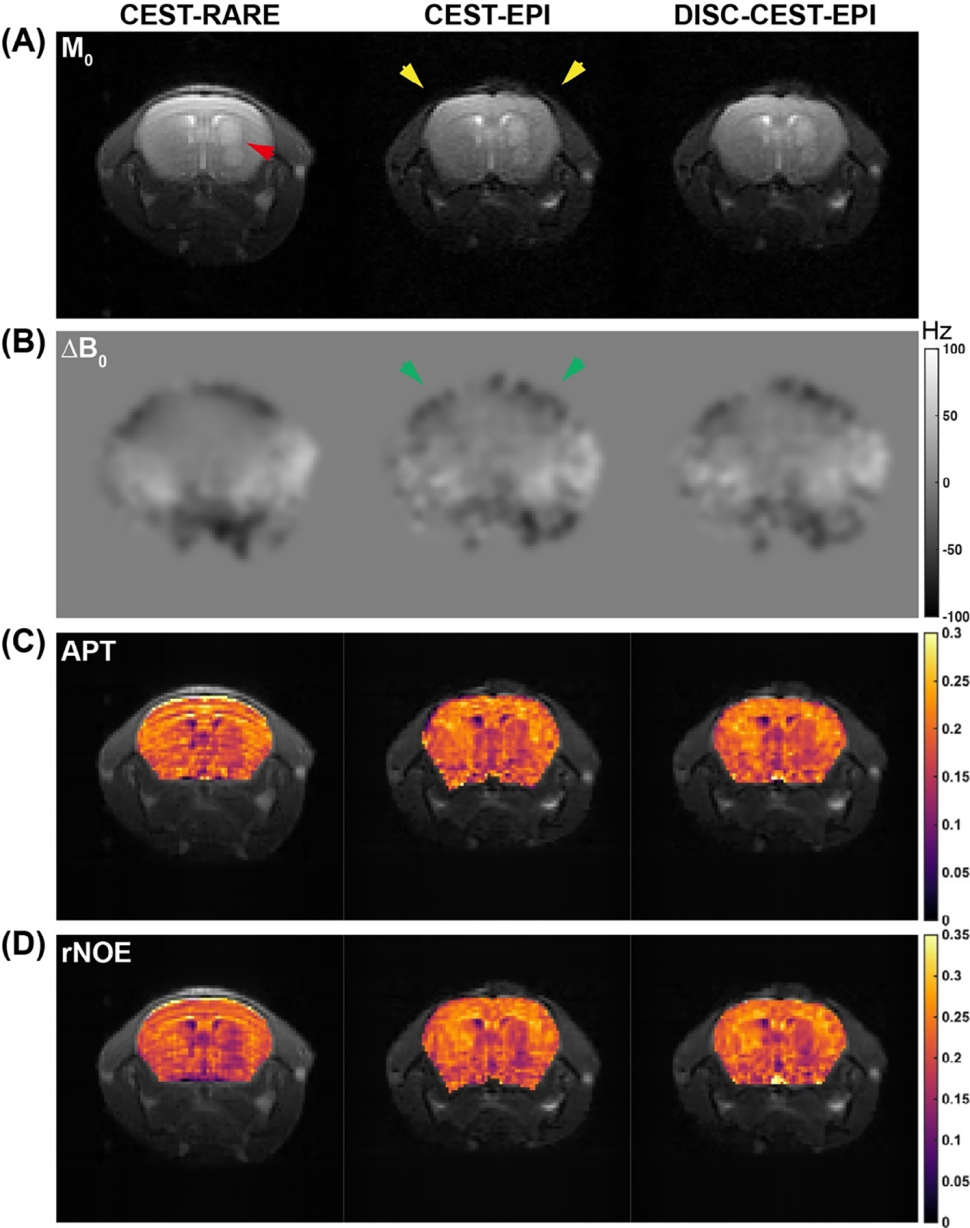
Comparison of CEST‐rapid acquisition with relaxation enhancement (RARE) with CEST‐EPI/distortion self‐correction (DISC)‐CEST‐EPI in a tumor mouse brain at 3 T. (A) Original CEST images (M_0_ images). (B) ΔB_0_ maps generated from Z‐spectrum. (C) Amide proton transfer (APT) maps. (D) Relayed nuclear Overhauser effects (rNOE) maps. Phase encoding is along vertical view.

**FIGURE 5:**
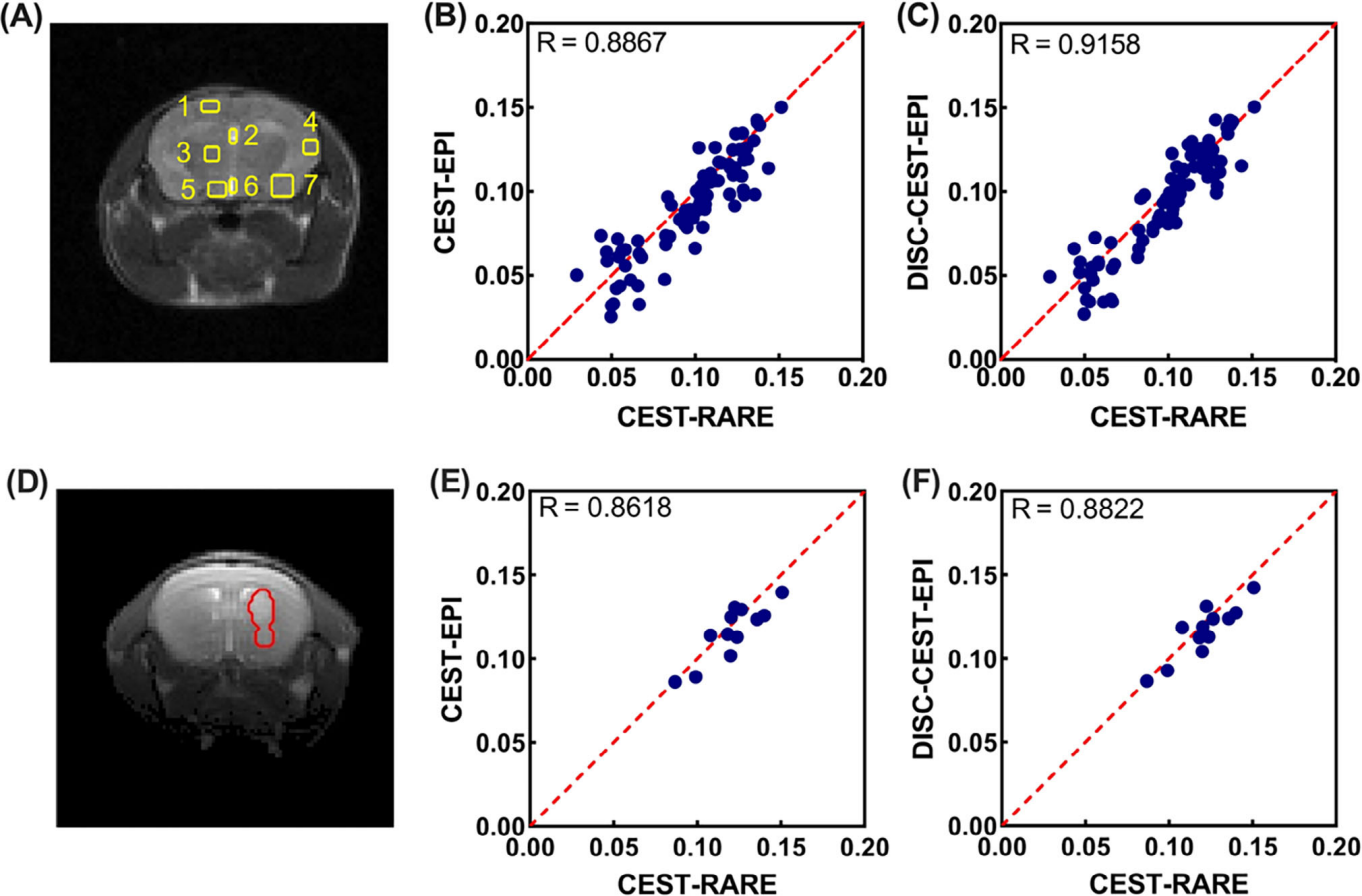
Spatial correlation of CEST values, including amide proton transfer (APT) and relayed nuclear Overhauser effects (rNOE) contrasts, between CEST‐rapid acquisition with relaxation enhancement (RARE) and CEST‐EPI/distortion self‐correction (DISC)‐CEST‐EPI in normal mice and tumor mice at 3 T. (A) Seven regions of interest in the mouse brain were selected for correlation analysis. (B) Correlation of CEST‐RARE with CEST‐EPI in normal mice. (C) Correlation of CEST‐RARE with DISC‐CEST‐EPI in normal mice. (D) Tumor region of mouse brain for correlation analysis. (E) Correlation of CEST‐RARE with CEST‐EPI in tumor mice. (F) Correlation of CEST‐RARE with DISC‐CEST‐EPI in tumor mice.

We then evaluated the performance of DISC‐CEST‐EPI in six mice with brain tumor (Figure 4). The tumor was implanted in the anterior part of the brain (indicated by red arrow in Figure 4A). Both rNOE and APT maps showed hypointensity in tumor regions compared to contralateral brain regions (Figure 4C,D). Additionally, DISC‐CEST‐EPI improved the SSIM value of the tumor CEST dataset (Table 1) from 0.800 ± 0.045 to 0.827 ± 0.045 (*p* = 0.0001). The SSIM values of the APT and rNOE maps increased from 0.913 ± 0.019 and 0.907 ± 0.020 to 0.933 ± 0.013 (*p* = 0.0082) and 0.930 ± 0.016 (*p* = 0.0024), respectively. Correlation analysis between CEST‐EPI/DISC‐CEST‐EPI and CEST‐RARE, using mean values of APT and rNOE extracted from seven regions of interest (ROIs) in the mouse brain (Figure 5A), showed that DISC‐CEST‐EPI exhibited better spatial consistency than CEST‐EPI with correlation coefficients of *R* = 0.8867 and 0.9158, respectively (Figure 5B,C). Correlation analysis between CEST‐EPI/DISC‐CEST‐EPI and CEST‐RARE, using mean values of APT and rNOE extracted from tumor regions in the mouse brain (Figure 5D), showed that DISC‐CEST‐EPI exhibited better spatial consistency than CEST‐EPI with correlation coefficients of *R* = 0.8618and 0.8822, respectively (Figure 5E,F). These findings supported the effectiveness of the DISC strategy in correcting geometric distortion for in vivo CEST MRI at 3 T.

### 3.3 The 11.7 T in vivo mouse brain results

**FIGURE 6:**
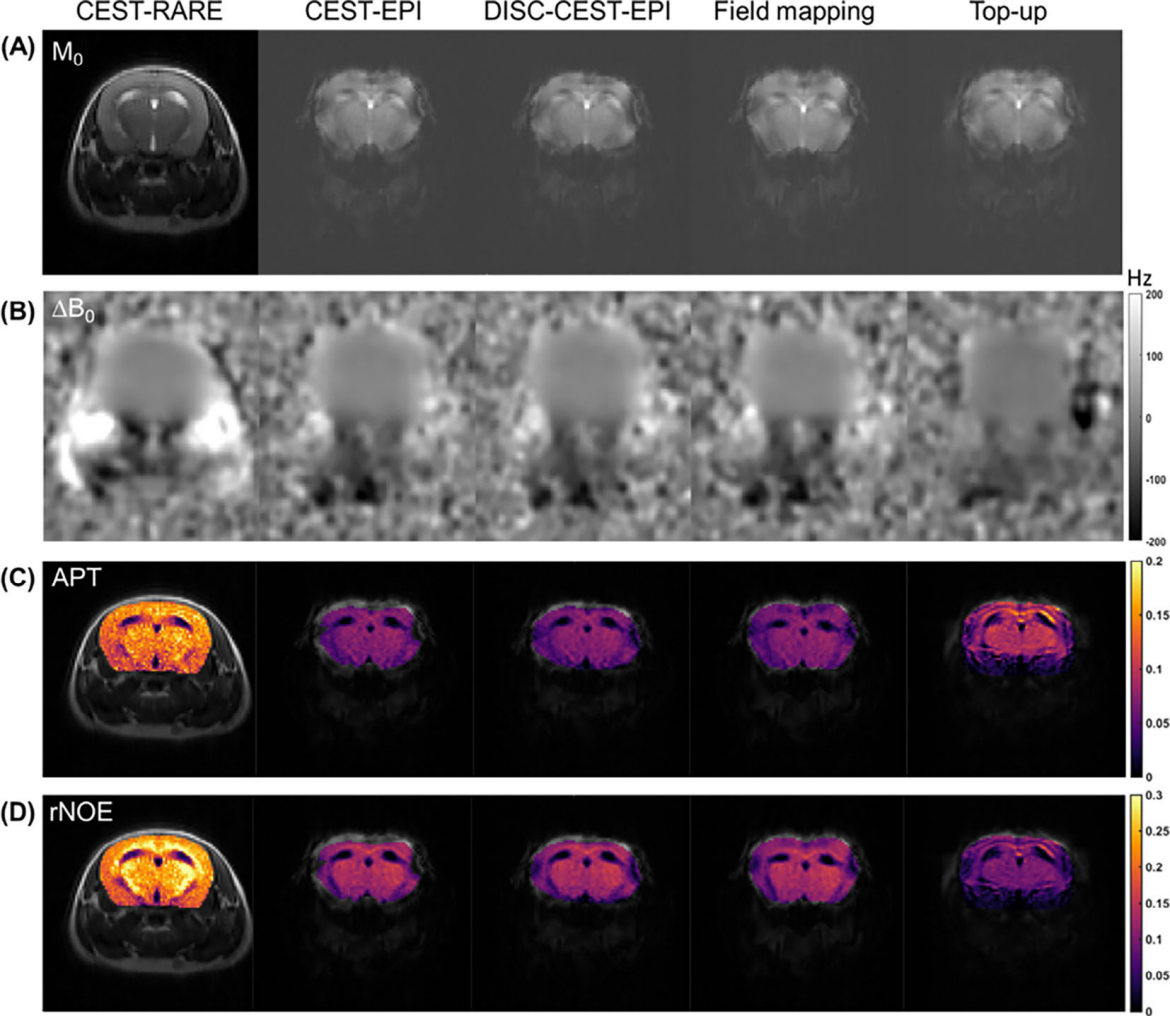
Comparison of CEST‐rapid acquisition with relaxation enhancement (RARE) with CEST‐EPI, distortion self‐correction (DISC)‐CEST‐EPI, field‐mapping, and top‐up in mouse brain at 11.7 T. (A) Original CEST images (M_0_ images). (B) ΔB_0_ maps generated from Z‐spectrum. (C) Amide proton transfer (APT) maps. (D) Relayed nuclear Overhauser effects (rNOE) maps. Phase encoding is along vertical view.

The results of three AQP4 heterozygotes mice are shown in Figure 6. CEST‐RARE image showed good quality of clear boundaries and high SNR, whereas CEST‐EPI suffered great distortion and extremely low SNR as evidenced by the almost disappeared muscle contrast. These problems persisted in all images after correction (Figure 6A). The mouse brain exhibited greater distortion on the edge region compared to other regions, with distortion being more pronounced than that in images obtained at 3 T (Figure 3A). From the results we can see the distortion could be corrected using DISC, field‐mapping and top‐up methods. When comparing ΔB_0_ map calculated from Z‐spectra (Figure 6B), CEST‐EPI, DISC‐CEST‐EPI, and field‐mapping method show similar results, whereas top‐up method exhibits lower ΔB_0_ values than other methods. Furthermore, none of the evaluated methods effectively produced a favorable ΔB_0_ map comparable to the one obtained at 3 T (Figure 3B). The high field strength poses challenges in correcting distortion using either the scanned or estimated ΔB_0_ map. This issue also affects the APT and rNOE maps extracted from Z‐spectrum (Figure 6C). Moreover, because of the extremely low SNR, the APT and rNOE maps of all CEST‐EPI methods are lower than those of CEST‐RARE (Figure 6C,D). Although CEST‐EPI, DISC‐CEST‐EPI, and field‐mapping method show similar CEST contrast levels for APT and rNOE, top‐up method exhibits a higher APT map and a lower rNOE map. The SSIM values validated these findings (Table 2). When using CEST‐RARE as a reference, the SSIM value of APT map increased from 0.854 (CEST‐EPI) to 0.859 (DISC‐CEST‐EPI), 0.862 (field‐mapping method) and 0.868 (top‐up method), the SSIM value of rNOE map increased from 0.847 (CEST‐EPI) to 0.853 (DISC‐CEST‐EPI), 0.850 (field‐mapping method) and 0.848 (top‐up method).

**TABLE 2:** Comparison of SSIM values of CEST images and contrast maps for CEST‐EPI, DISC‐CEST‐EPI, field‐mapping, and top‐up of mice at 11.7 T.

	Mouse no.	CEST‐EPI	DISC‐CEST‐EPI	Field‐mapping	Top‐up
All CEST images	1	0.471	0.466	0.453	0.457
	2	0.480	0.466	0.460	0.465
	3	0.541	0.540	0.523	0.543
	Mean	0.497	0.491	0.479	0.483
APT map	1	0.869	0.873	0.877	0.897
	2	0.839	0.843	0.849	0.845
	3	0.854	0.861	0.861	0.862
	Mean	0.854	0.859	0.862	0.868
rNOE map	1	0.862	0.866	0.864	0.861
	2	0.833	0.842	0.839	0.838
	3	0.845	0.851	0.846	0.846
	Mean	0.847	0.853	0.850	0.848

Abbreviations: APT, amide proton transfer; DISC, distortion self‐correction; rNOE, relayed nuclear Overhauser effects; SSIM, structural similarity index measure.

## 4 DISCUSSION

In this study, we investigated the effectiveness of distortion self‐correction strategy for single‐shot EPI. In vitro and in vivo results demonstrated that the DISC‐CEST‐EPI outperformed CEST‐EPI in both quantitative and qualitative comparison, providing evidence that ΔB_0_ field map generated from Z‐spectra can be used to correct distortion in CEST‐EPI. Although new methods such as using deep learning to directly generate CEST maps without calculating ΔB_0_ map are acceptable, most researchers still use ΔB_0_ map generated from Z‐spectra to do the ΔB_0_ shift correction first before performing further postprocessing. Therefore, ΔB_0_ map generation is a necessary intermediate step for Z‐spectrum analysis. Here, we further extend the utilization of ΔB_0_ map for correcting the geometric distortion without the need for additional acquisition of a ΔB_0_ map. Single‐shot EPI is commonly used in CEST MRI studies to achieve fast CEST acquisition. However, it is susceptible to geometric distortion induced by ΔB_0_ inhomogeneity. This study demonstrates that using the ΔB_0_ map generated from Z‐spectra to correct the distortion artifact caused by EPI appears to be a promising approach.

For the phantom experiment conducted at 3 T (Figure 2), the distortion level was clearly aligned with ΔB_0_ value along y direction, which was caused by phase encoding, while no visible shift could be observed on *x* direction. The phantom pattern is regular and the phantom ingredients are simple (water and creatine), leading to better performance in distortion correction compared to mouse study (Figures 3 and 4). This also results in higher SSIM values in phantom than those in mouse brains. The phantom DISC‐CEST‐EPI almost entirely corrected the geometrical distortion, whereas the mouse DISC‐CEST‐EPI still exhibited some remaining distortion, especially at interfaces with high ΔB_0_ values. Nevertheless, the DISC‐CEST‐EPI effectively corrected the distortion within the brain region where the ΔB_0_ map was less in homogeneous than other interfaces. Regarding the correlation results, the phantom was specifically designed to create a gradient of creatine concentration in different test tubes, which resulted in higher correlation coefficient values compared to those obtained from the ROI analysis in the mouse brain. Nonetheless, DISC‐CEST‐EPI in both the phantom and mice showed improved SSIM of CEST maps generated using MTR_asym_ and higher spatial consistency of CEST values compared to CEST‐EPI, indicating the effectiveness and applicability of the distortion self‐correction strategy.

Interestingly, four tumor mice imaged using the surface coil (mice 9–12) showed higher SSIM values in all CEST source images compared to other mice imaged using the volume coil (mice 1–8), as shown in Table 1. This is because the images acquired by the surface coil have a higher SNR for proximal brain region and a lower SNR for distal bottom muscle region (Figures 4 and 5D–F), resulting in a smaller head region involved in SSIM calculation. Nevertheless, DISC‐CEST‐EPI improved SSIM compared with CEST‐EPI regardless of the receiving coil. Moreover, the SSIM improvement was observed in APT and rNOE maps of all investigated mice. In terms of spatial consistency analysis, normal mice and tumor mice exhibited similar correlation coefficient values from selected ROIs (Figure 5D–F). Although DISC‐CEST‐EPI of each mouse type showed improved SSIM for all CEST images and CEST maps, along with higher spatial consistency of CEST values compared to CEST‐EPI, it did not exhibit noticeable contrast difference between tumor region and contralateral brain region. This is because the ΔB_0_ inhomogeneity in the central brain region is relatively small when compared to the limbic brain region, causing a small structural distortion in the central brain region. From another perspective, the distortion self‐correction process will not notably alter the CEST contrast if the distortion in a certain area is negligible.

In experiments conducted at 11.7 T, CEST‐RARE demonstrated good image quality. However, CEST‐EPI exhibited severe distortion and extremely low SNR. This was attributed to the ultra‐short T_2_ at 11.7 T,^62^ resulting in only the brain region being visible. Even with the application of distortion correction methods, the inherent issue stemming from the acquisition parameters cannot be eliminated. Among the three correction methods, DISC‐CEST‐EPI exhibited higher residual distortion compared to field‐mapping and top‐up methods. Field‐mapping method provided superior distortion correction by using an accurately acquired ΔB_0_ map instead of an estimated ΔB_0_ map like the other two methods. Top‐up method achieved the most effective compression correction by acquiring two EPI images with opposite phase encodings, allowing it to use both extension and compression results for correction, which is crucial for addressing pixel intensity accumulation issue. This effect was also evident in the APT map and rNOE map, as top‐up method exhibited the most comparable structure compared to the other two methods, with the most noticeable intensity changes. However, the disadvantage of top‐up is that it doubles the scanning time. Because of the low SNR level, all these three correction methods showed lower CEST contrast than CEST‐RARE although the regional contrast trend is similar within the brain regions. The CEST contrast levels of CEST‐EPI and the corrected images are approximately half of those in CEST‐RARE images, making it difficult to conduct the correlation analysis. All three methods exhibited higher SSIM values than CEST‐EPI in APT maps and rNOE maps, but lower SSIM values in all CEST source images. This discrepancy can be attributed to the fact that the SSIM of the former two types of maps was calculated from the brain region only, whereas the SSIM of the CEST source images was calculated from the entire head region where muscle signal almost disappeared. Nevertheless, current results validate that DISC‐CEST‐EPI can achieve distortion correction at ultra‐high field, but with limited performance. Although the performance of DISC‐CEST‐EPI is barely comparable enough with existing methods such as field‐mapping and top‐up methods at ultra‐high field (11.7 T), its outcomes are not as desirable as those achieved at 3 T.

Based on our experiments conducted at both 3 T and 11.7 T, DISC‐CEST‐EPI has been verified as a viable method for distortion correction in CEST‐EPI. However, its effectiveness may diminish as distortion levels increase with higher field strengths. Because DISC‐CEST‐EPI requires the ΔB_0_ map to achieve the correction, the accuracy of estimating ΔB_0_ map from Z‐spectrum is very decisive for correction performance. The estimation of ΔB_0_ map uses minimum search, which is determined by the Z‐spectrum value at approximately 0 ppm and the ΔB_0_ value is usually within a small range from −1 ppm to 1 ppm. To ensure the generation of an accurate ΔB_0_ map from the Z‐spectrum, we suggest acquiring as many offsets as feasible between −1 ppm to 1 ppm. This will enhance the accuracy of the resulting ΔB_0_ map, but at the cost of increasing the scanning time. The advantage of DISC method is that it eliminates the need for additional scans, whereas the other two methods require additional acquisition. For example, in the experiment at 11.7 T, the field‐mapping method involved acquiring a ΔB0 map using a two‐TE method, which took approximately 1.3 minutes. This time frame is equivalent to conducting a 13‐offset scan in CEST‐EPI. The offset number is higher than the typical number of scans used for estimating a ΔB0 map based on minimum search in Z‐spectra. Recent advancements in acceleration techniques have enabled rapid acquisition of ΔB_0_ maps for field‐mapping method,^63^ potentially allowing for additional maps to be obtained without significantly increasing scan time.

Despite its feasibility, there are still some limitations of DISC‐CEST‐EPI. When facing severe distortions at high field strengths with high B_0_ inhomogeneity, the correction performance of DISC‐CEST‐EPI could be compromised. The estimated ΔB_0_ map itself suffers from distortion issues, resulting in a lack of ΔB_0_ information, especially in boundary regions. Proper methods to retrospectively estimate ΔB_0_ map together with optimized correction algorithms based on the specific case of DISC‐CEST‐EPI is a potential solution that is worth future investigation. For example, we propose that postprocessing steps, such as ΔB_0_ map smoothing or advanced interpolation to achieve smaller ΔB_0_ variation and better estimation of the lost information to mitigate these issues in future implementations, enhance the robustness of DISC strategy. However, limitations such as pixel intensity accumulation cannot be solved by DISC‐CEST‐EPI or field‐mapping; instead, they can be addressed using the top‐up method, which requires dual scans with opposite phase encoding directions. The dual scans enhance the SNR and correction accuracy of the top‐up method, however, they also result in a doubling of the scan time. In addition, top‐up can correct for dynamic changes in B_0_ inhomogeneity better than DISC‐CEST‐EPI. Selecting the appropriate correction method requires careful consideration of several critical factors, including magnetic field strength, scan time, the severity of ΔB_0_ inhomogeneity, CEST protocol, and the target accuracy and robustness. Our findings suggest that DISC is the most time‐efficient method for CEST‐EPI at clinical field strengths like 3 T, providing effective correction for moderate B_0_ inhomogeneity without the need for additional scans. DISC is recommended when full Z‐spectra are acquired. However, for CEST studies that do not acquire full Z‐spectra, such as 3‐point and 7‐point APTw MRI protocols,^64^ the field‐mapping method is suggested. This approach provides B_0_ inhomogeneity information, but requires an additional scan. The top‐up method is particularly recommended at ultra‐high magnetic fields, where severe B_0_ inhomogeneity is common. It is considered the current gold standard for applications requiring maximum geometric fidelity, as it uniquely corrects for signal pile‐up. However, implementing the top‐up method doubles the scan time of CEST‐EPI, so its use is recommended only when sufficient total scan time is available. Future research could integrate other distortion correction methods to tackle this issue while mitigating the potential increase in scan time.

## 5 CONCLUSIONS

CEST‐EPI is a rapid imaging technique, which is prone to image distortion resulting from field inhomogeneities. In this study, we evaluated the effectiveness of using field map generated by Z‐spectra to achieve self‐correction of distortion in single‐shot CEST‐EPI, eliminating the need for additional field map acquisition. Our results, obtained from phantom and mouse brain experiments, demonstrated that DISC‐CEST‐EPI effectively corrected the distortion in CEST‐EPI at 3 T MRI, therefore, enhancing the SSIM and CEST quantification. This self‐correction strategy for distortion has the potential to enhance the image quality and improve diagnostic accuracy when using single‐shot EPI for accelerated CEST acquisition at low‐field MRI. However, the performance of DISC‐CEST‐EPI at ultra‐high fields is currently limited, and further development is necessary to enhance its functionality in the future.

## Data Availability

The code and exemplary data that support the findings of this study are openly available at https://github.com/JianpanHuang/DISC‐CEST‐EPI.
